# 1-(3-Amino-1*H*-inden-2-yl)ethanone

**DOI:** 10.1107/S1600536808033485

**Published:** 2008-10-31

**Authors:** Dong-Yue Hu, Zhi-Rong Qu

**Affiliations:** a Ordered Matter Science Research Center, College of Chemistry and Chemical Engineering, Southeast University, Nanjing 210096, People’s Republic of China

## Abstract

The title compound, C_11_H_11_NO, was synthesized by the reaction of 2-(bromo­meth­yl)benzonitrile and acetyl­acetone in the presence of KOH. In the crystal packing, mol­ecules are linked by inter­molecular N—H⋯O hydrogen bonds into chains running parallel to the *b* axis. Centrosymmetrically-related chains inter­act further through weak C—H⋯π inter­actions.

## Related literature

For the crystal structures of related compounds, see: Choi *et al.* (1999 ▶); Fu & Zhao (2007 ▶).
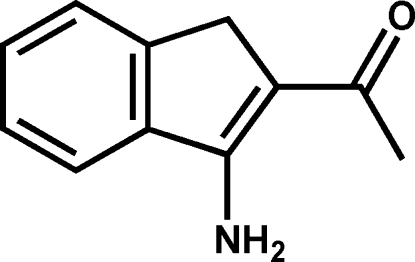


## Experimental

### 

#### Crystal data

C_11_H_11_NO
*M* *_r_* = 173.21
Monoclinic, 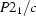

*a* = 8.1794 (4) Å
*b* = 10.6905 (5) Å
*c* = 10.5602 (6) Å
β = 93.783 (8)°
*V* = 921.39 (8) Å^3^
*Z* = 4
Mo *K*α radiation
μ = 0.08 mm^−1^
*T* = 293 (2) K
0.25 × 0.16 × 0.14 mm

#### Data collection

Rigaku SCXmini diffractometer
Absorption correction: multi-scan (*CrystalClear*; Rigaku, 2005 ▶) *T*_min_ = 0.980, *T*_max_ = 0.989
9369 measured reflections
2108 independent reflections
1385 reflections with *I* > 2σ(*I*)
*R*_int_ = 0.049

#### Refinement

*R*[*F* ^2^ > 2σ(*F* ^2^)] = 0.060
*wR*(*F* ^2^) = 0.171
*S* = 1.04
2108 reflections
119 parameters
H-atom parameters constrained
Δρ_max_ = 0.21 e Å^−3^
Δρ_min_ = −0.20 e Å^−3^

### 

Data collection: *CrystalClear* (Rigaku, 2005 ▶); cell refinement: *CrystalClear*; data reduction: *CrystalClear*; program(s) used to solve structure: *SHELXS97* (Sheldrick, 2008 ▶); program(s) used to refine structure: *SHELXL97* (Sheldrick, 2008 ▶); molecular graphics: *SHELXTL* (Sheldrick, 2008 ▶); software used to prepare material for publication: *SHELXL97* and *PRPKAPPA* (Ferguson, 1999 ▶).

## Supplementary Material

Crystal structure: contains datablocks global, I. DOI: 10.1107/S1600536808033485/rz2252sup1.cif

Structure factors: contains datablocks I. DOI: 10.1107/S1600536808033485/rz2252Isup2.hkl

Additional supplementary materials:  crystallographic information; 3D view; checkCIF report

## Figures and Tables

**Table 1:** Hydrogen-bond geometry (Å, °) *Cg*1 is the centroid of the C7–C11/C13 ring.

*D*—H⋯*A*	*D*—H	H⋯*A*	*D*⋯*A*	*D*—H⋯*A*
N1—H1*A*⋯O2	0.86	2.17	2.766 (2)	126
N1—H1*B*⋯O2^i^	0.86	2.09	2.924 (2)	164
C2—H2*B*⋯*Cg*1^ii^	0.97	2.77	3.631 (2)	148

Symmetry codes: (i) 
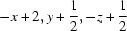
; (ii) 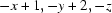.

**Fig. 1.**
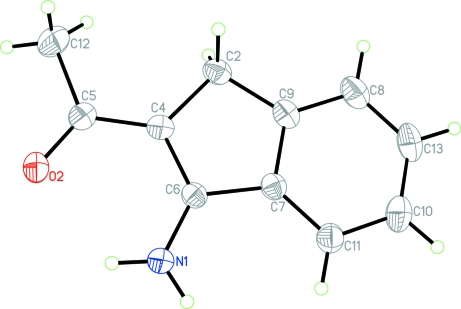
The molecular structure of the title compound with the atom-numbering scheme. Displacement ellipsoids are drawn at the 30% probability level.

**Fig. 2.**
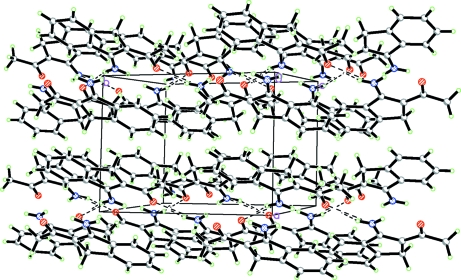
Packing diagram of the title compound. Intermolecular hydrogen bonds are shown as dashed lines.

## supplementary crystallographic information

### Comment

In recent years, the synthesis and characterization of new ligands containing amino donor groups has received considerable attention due to the potential applications in coordination chemistry (Choi *et al.*, 1999; Fu & Zhao, 2007). We report here the crystal structure of the title compound, which was obtained by the reaction of *o*-(bromomethyl)benzonitrile and acetylacetone in the presence of KOH.

In the molecule of the title compound (Fig. 1), the five-membered ring formed through the reaction is planar, and the geometric parameters are in the usual ranges. The molecular conformation is stabilized by an intramolecular N—H···O hydrogen bond (Table 1). In the crystal structure (Fig. 2), molecules are connected by intermolecular N—H···O hydrogen bonds into chains running parallel to the *b* axis (Table 1). Centrosymmetrically-related chains are further interacting through weak C—H···π interactions (Table 1).

### Experimental

Acetylacetone (0.5 g, 0.5 mmol) and *o*-(bromomethyl)-benzonitrile (0.98 g, 0.5 mmol) were dissolved in methanol (30 ml) in the presence of KOH (0.28 g, 0.5 mmol) and the mixture refluxed for 24 h at 393K. After cooling to room temperature, most of the solvent was removed by vacuum filtration. Colourless crystals of the title compound suitable for X-ray diffraction analysis were obtained by slow evaporation of the remaining solvent.

### Refinement

All H atoms were placed at calculated positions and allowed to ride on their parent atoms, with C—H = 0.93–0.97 Å, N—H = 0.86 Å, and with U_iso_(H) = 1.2U_eq_(C, N) or 1.5U_eq_(C) for methyl H atoms.

### Crystal data

**Table:**

C_11_H_11_NO	*F*(000) = 368
*M**_r_* = 173.21	*D*_x_ = 1.249 Mg m^−^^3^
Monoclinic, *P*2_1_/*c*	Mo *K*α radiation, λ = 0.71073 Å
Hall symbol: -P 2ybc	Cell parameters from 4430 reflections
*a* = 8.1794 (4) Å	θ = 3.1–27.4°
*b* = 10.6905 (5) Å	µ = 0.08 mm^−^^1^
*c* = 10.5602 (6) Å	*T* = 293 K
β = 93.783 (8)°	Block, colourless
*V* = 921.39 (8) Å^3^	0.25 × 0.16 × 0.14 mm
*Z* = 4	

### Data collection

**Table:**

Rigaku SCXmini diffractometer	2108 independent reflections
Radiation source: fine-focus sealed tube	1385 reflections with *I* > 2σ(*I*)
graphite	*R*_int_ = 0.049
Detector resolution: 13.6612 pixels mm^-1^	θ_max_ = 27.5°, θ_min_ = 3.1°
CCD profile fitting scans	*h* = −10→10
Absorption correction: multi-scan (CrystalClear; Rigaku, 2005)	*k* = −13→13
*T*_min_ = 0.980, *T*_max_ = 0.989	*l* = −13→13
9369 measured reflections	

### Refinement

**Table:**

Refinement on *F*^2^	Primary atom site location: structure-invariant direct methods
Least-squares matrix: full	Secondary atom site location: difference Fourier map
*R*[*F*^2^ > 2σ(*F*^2^)] = 0.060	Hydrogen site location: inferred from neighbouring sites
*wR*(*F*^2^) = 0.171	H-atom parameters constrained
*S* = 1.04	*w* = 1/[σ^2^(*F*_o_^2^) + (0.0814*P*)^2^ + 0.2011*P*] where *P* = (*F*_o_^2^ + 2*F*_c_^2^)/3
2108 reflections	(Δ/σ)_max_ < 0.001
119 parameters	Δρ_max_ = 0.21 e Å^−^^3^
0 restraints	Δρ_min_ = −0.20 e Å^−^^3^

### Special details

**Table:**

Geometry. All e.s.d.'s (except the e.s.d. in the dihedral angle between two l.s. planes) are estimated using the full covariance matrix. The cell e.s.d.'s are taken into account individually in the estimation of e.s.d.'s in distances, angles and torsion angles; correlations between e.s.d.'s in cell parameters are only used when they are defined by crystal symmetry. An approximate (isotropic) treatment of cell e.s.d.'s is used for estimating e.s.d.'s involving l.s. planes.
Refinement. Refinement of *F*^2^ against ALL reflections. The weighted *R*-factor *wR* and goodness of fit *S* are based on *F*^2^, conventional *R*-factors *R* are based on *F*, with *F* set to zero for negative *F*^2^. The threshold expression of *F*^2^ > σ(*F*^2^) is used only for calculating *R*-factors(gt) *etc*. and is not relevant to the choice of reflections for refinement. *R*-factors based on *F*^2^ are statistically about twice as large as those based on *F*, and *R*- factors based on ALL data will be even larger.

### Fractional atomic coordinates and isotropic or equivalent isotropic displacement parameters (Å^2^)

**Table:**

	*x*	*y*	*z*	*U*_iso_*/*U*_eq_	
C2	0.6932 (2)	0.90032 (19)	−0.07286 (18)	0.0447 (5)	
H2A	0.7446	0.8647	−0.1448	0.054*	
H2B	0.5859	0.8630	−0.0672	0.054*	
C4	0.7981 (2)	0.88190 (18)	0.04815 (17)	0.0393 (5)	
C5	0.8500 (2)	0.76630 (18)	0.1034 (2)	0.0434 (5)	
C6	0.8406 (2)	0.99769 (18)	0.10065 (17)	0.0363 (4)	
C7	0.7680 (2)	1.09672 (19)	0.01929 (18)	0.0389 (5)	
C8	0.5992 (3)	1.1144 (2)	−0.1751 (2)	0.0584 (6)	
H8	0.5401	1.0783	−0.2440	0.070*	
C9	0.6815 (2)	1.0401 (2)	−0.08348 (18)	0.0439 (5)	
C10	0.6928 (3)	1.2986 (3)	−0.0592 (2)	0.0629 (7)	
H10	0.6952	1.3853	−0.0521	0.076*	
C11	0.7750 (3)	1.2260 (2)	0.0329 (2)	0.0490 (6)	
H11	0.8332	1.2626	0.1019	0.059*	
C12	0.7998 (3)	0.6463 (2)	0.0355 (3)	0.0667 (7)	
H12A	0.8792	0.5824	0.0570	0.100*	
H12B	0.7939	0.6602	−0.0545	0.100*	
H12C	0.6945	0.6204	0.0608	0.100*	
C13	0.6070 (3)	1.2432 (2)	−0.1617 (2)	0.0664 (7)	
H13	0.5533	1.2936	−0.2231	0.080*	
N1	0.9314 (2)	1.01839 (16)	0.20784 (15)	0.0490 (5)	
H1A	0.9702	0.9564	0.2522	0.059*	
H1B	0.9512	1.0938	0.2328	0.059*	
O2	0.93402 (18)	0.75969 (13)	0.20655 (14)	0.0527 (4)	

### Atomic displacement parameters (Å^2^)

**Table:**

	*U*^11^	*U*^22^	*U*^33^	*U*^12^	*U*^13^	*U*^23^
C2	0.0439 (11)	0.0511 (13)	0.0384 (11)	0.0004 (9)	−0.0018 (8)	−0.0056 (9)
C4	0.0376 (10)	0.0419 (11)	0.0383 (10)	−0.0022 (8)	0.0014 (8)	−0.0035 (8)
C5	0.0434 (11)	0.0401 (12)	0.0470 (12)	−0.0014 (9)	0.0046 (9)	−0.0010 (8)
C6	0.0332 (9)	0.0403 (11)	0.0354 (10)	−0.0004 (8)	0.0023 (7)	0.0010 (8)
C7	0.0346 (9)	0.0424 (11)	0.0399 (11)	0.0010 (8)	0.0036 (8)	0.0024 (8)
C8	0.0553 (13)	0.0740 (18)	0.0446 (13)	0.0109 (12)	−0.0053 (10)	0.0031 (11)
C9	0.0392 (10)	0.0544 (14)	0.0380 (11)	0.0049 (9)	0.0023 (8)	0.0025 (9)
C10	0.0714 (16)	0.0487 (13)	0.0685 (17)	0.0096 (12)	0.0037 (13)	0.0164 (12)
C11	0.0484 (12)	0.0456 (14)	0.0526 (12)	0.0016 (10)	0.0007 (9)	0.0042 (10)
C12	0.0854 (18)	0.0415 (13)	0.0719 (16)	−0.0014 (12)	−0.0041 (13)	−0.0100 (11)
C13	0.0691 (16)	0.0700 (17)	0.0593 (15)	0.0214 (13)	−0.0016 (12)	0.0218 (12)
N1	0.0605 (11)	0.0381 (9)	0.0461 (10)	−0.0026 (8)	−0.0137 (8)	0.0000 (7)
O2	0.0660 (10)	0.0415 (8)	0.0493 (9)	0.0033 (7)	−0.0075 (7)	0.0062 (6)

### Geometric parameters (Å, °)

**Table:**

C2—C9	1.501 (3)	C8—C9	1.391 (3)
C2—C4	1.505 (3)	C8—H8	0.9300
C2—H2A	0.9700	C10—C11	1.383 (3)
C2—H2B	0.9700	C10—C13	1.384 (3)
C4—C6	1.391 (3)	C10—H10	0.9300
C4—C5	1.420 (3)	C11—H11	0.9300
C5—O2	1.251 (2)	C12—H12A	0.9600
C5—C12	1.513 (3)	C12—H12B	0.9600
C6—N1	1.331 (2)	C12—H12C	0.9600
C6—C7	1.464 (3)	C13—H13	0.9300
C7—C11	1.391 (3)	N1—H1A	0.8600
C7—C9	1.394 (3)	N1—H1B	0.8600
C8—C13	1.385 (4)		
			
C9—C2—C4	102.92 (15)	C8—C9—C7	119.4 (2)
C9—C2—H2A	111.2	C8—C9—C2	130.2 (2)
C4—C2—H2A	111.2	C7—C9—C2	110.33 (17)
C9—C2—H2B	111.2	C11—C10—C13	120.5 (3)
C4—C2—H2B	111.2	C11—C10—H10	119.7
H2A—C2—H2B	109.1	C13—C10—H10	119.7
C6—C4—C5	123.41 (18)	C10—C11—C7	118.1 (2)
C6—C4—C2	109.61 (17)	C10—C11—H11	121.0
C5—C4—C2	126.98 (18)	C7—C11—H11	121.0
O2—C5—C4	122.65 (18)	C5—C12—H12A	109.5
O2—C5—C12	118.77 (19)	C5—C12—H12B	109.5
C4—C5—C12	118.6 (2)	H12A—C12—H12B	109.5
N1—C6—C4	126.71 (18)	C5—C12—H12C	109.5
N1—C6—C7	124.11 (17)	H12A—C12—H12C	109.5
C4—C6—C7	109.18 (16)	H12B—C12—H12C	109.5
C11—C7—C9	121.81 (19)	C10—C13—C8	121.5 (2)
C11—C7—C6	130.23 (19)	C10—C13—H13	119.2
C9—C7—C6	107.96 (18)	C8—C13—H13	119.2
C13—C8—C9	118.7 (2)	C6—N1—H1A	120.0
C13—C8—H8	120.7	C6—N1—H1B	120.0
C9—C8—H8	120.7	H1A—N1—H1B	120.0

### Hydrogen-bond geometry (Å, °)

**Table:**

*D*—H···*A*	*D*—H	H···*A*	*D*···*A*	*D*—H···*A*
N1—H1A···O2	0.86	2.17	2.766 (2)	126
N1—H1B···O2^i^	0.86	2.09	2.924 (2)	164
C2—H2B···Cg1^ii^	0.97	2.77	3.631 (2)	148

Symmetry codes: (i) −*x*+2, *y*+1/2, −*z*+1/2; (ii) −*x*+1, −*y*+2, −*z*.
